# Mycophenolate Mofetil Modulates Differentiation of Th1/Th2 and the Secretion of Cytokines in an Active Crohn’s Disease Mouse Model

**DOI:** 10.3390/ijms161125985

**Published:** 2015-11-06

**Authors:** Qing-Kang Lv, Ju-Xiong Liu, Su-Nan Li, Ying-Jie Gao, Yan Lv, Zi-Peng Xu, Bing-Xu Huang, Shi-Yao Xu, Dong-Xue Yang, Ya-Long Zeng, Dian-Feng Liu, Wei Wang

**Affiliations:** 1 College of Veterinary Medicine, Jilin University, China; lvqingkang@hotmail.com (Q.-K.L.); juxiong@jlu.edu.cn (J.-X.L.); fengerwin1@sina.com (S.-N.L.); yjgao@jlu.edu.cn (Y.-J.G.); lvyanty@jlu.edu.cn (Y.L.); huangbingxu123@outlook.com (B.-X.H.); gladys20@sina.com (S.-Y.X.); yangdxaig@outlook.com (D.-X.Y.); zengyalong@foxmail.com (Y.-L.Z.); liudf@jlu.edu.cn (D.-F.L.); 2 PengCheng Gastroenterology Hospital, Changchun 130062, China; xuzipeng1950@sina.com

**Keywords:** mycophenolate mofetil, Th1/Th2 cells, cytokine, inflammatory bowel disease

## Abstract

Mycophenolate mofetil (MMF) is an alternative immunosuppressive agent that has been reported to be effective and well tolerated for the treatment of refractory inflammatory bowel disease (IBD). The aim of this study was to investigate the therapeutic effect of MMF on intestinal injury and tissue inflammation, which were caused by Crohn’s disease (CD). Here, trinitrobenzene sulfonic acid-relapsing (TNBS) colitis was induced in mice; then, we measured the differentiation of Th1/Th2 cells in mouse splenocytes by flow cytometry and the secretion of cytokines in mice with TNBS-induced colitis by real-time polymerase chain reaction and/or enzyme-linked immunosorbent assay (RT-PCR/ELISA). The results show that MMF significantly inhibited mRNA expression of pro-inflammatory cytokines IFN-γ, TNF-α, IL-12, IL-6, and IL-1β in mice with TNBS-induced colitis; however, MMF did not inhibit the expression of IL-10 mRNA. Additionally, ELISA showed that the serum levels of IFN-γ, TNF-α, IL-12, IL-6, and IL-1β were down-regulated in a TNBS model of colitis. Flow cytometric analysis showed MMF markedly reduced the percentages of Th1 and Th2 splenocytes in the CD mouse model. Mycophenolic acid (MPA) also significantly decreased the percentages of splenic Th1 and Th2 cells *in vitro*. Furthermore, MMF treatment not only significantly ameliorated diarrhea, and loss of body weight but also abrogated the histopathologic severity and inflammatory response of inflammatory colitis, and increased the survival rate of TNBS-induced colitic mice. These results suggest that treatment with MMF may improve experimental colitis and induce inflammatory response remission of CD by down-regulation of pro-inflammatory cytokines via modulation of the differentiation of Th1/Th2 cells.

## 1. Introduction

Inflammatory bowel disease (IBD) is a chronic, relapsing inflammatory disorder of the digestive tract that, comprises Crohn’s disease (CD) and ulcerative colitis (UC). It is characterized by dysregulated immune responses, altered cytokine production, and cellular inflammation, which ultimately lead to damage of the distal small intestine and the colonic mucosa [1]. Currently, the exact etiology of IBD is complicated and remains unknown, but it is related to many factors such as immunity, heredity, intestinal environment, and bacterial infection. Specifically, immunological factors have been found to play particularly important roles in the pathogenesis and sustainable development of inflammatory bowel disease. It is known that in UC inflammation is confined to the mucosal layer of the colon and spreads diffusely, whereas in CD, the inflammation is transmural and has a segmental distribution that can affect the entire gastrointestinal tract [2]. Studies have suggested that dysfunctional regulation of the immune system and increased T-cell-mediated responses to the microbial flora of the gastrointestinal tract appear to be involved [3,4]. Recently, the hypothesis that an imbalance of the CD4^+^ T subtypes (Thl/Th2) cells leads to pro-inflammatory/anti-inflammatory cytokine disproportionality and immune abnormalities was widely accepted to play an important role in the pathogenesis of IBD. Previous studies have suggested that CD is a T helper (Th)1–mediated disease, that is characterized by increased IFN-γ, TNF-α and IL-12 production, while UC is a Th2-mediated disease characterized by increased IL-6 and/or reduced IL-10 production. Furthermore, stimulated cells in the inflamed mucosa produce increased amounts of IFN-γ, IL-2, and IL-12 and reduced amounts of IL-4 and IL-10 [5,6]. These findings established a paradigm that links a Th1 response and a Th2 response to the pathogenesis of CD and UC, respectively. Increasing numbers of disease states have been analyzed on the basis of these Thl/Th2 cytokine secretion profiles, but with mixed results. Fuss *et al.*, hypothesized the pathogenesis of IBD is ultimately “channeled” into these effector T-cell pathways, and that we can treat these diseases by focusing on the cytokines that control these mechanisms [7]. Therefore, it is widely accepted that an imbalance of pro- and anti-inflammatory mediators is a key factor in the pathogenesis of IBD [8] and particularly in CD. For these reasons, alternative immune modulatory drugs (e.g., corticosteroids, infliximab and steroids) have been frequently prescribed for many decades for the treatment of active IBD. However, efficiency for the maintenance of remission is limited and the irreversible and severe side effects should not be ignored.

Mycophenolate mofetil (MMF), the 2,4-morpholino ethyl ester of mycophenolic acid, is a new immunosuppressive drug that has demonstrated considerable efficacy in allograft transplant recipients and in patients with a variety of autoimmune disorders [9,10,11,12,13]. In the liver, MMF is converted into mycophenolic acid (MPA), which is active form of MMF [14]. Mycophenolic acid is a non-competitive reversible inhibitor of type-2 inosine monophosphate dehydrogenase, which is a key enzyme in the *de novo* pathway of purine synthesis. MMF can alternatively suppress the proliferation of both B and T cells because unlike other cell types that can recycle nucleotides via a salvage pathway, B and T lymphocytes depend solely on *de novo* nucleotide synthesis. Therefore, the action of MMF is thought to suppress proliferation of this specific cell group. MMF decreases the recruitment of lymphocytes and monocytes, and consequently reduces the production of TNF-α and IL-1β into inflammatory sites *in vivo* [15,16,17]. Furthermore, a growing number of clinical reports showed the efficacy of this drug in the suppression of chronic inflammatory disorders and autoimmune disease, such as rheumatoid arthritis [15], pemphigus vulgaris [16], and psoriasis [18]. The use of MMF in IBD patients especially in those who are steroid-dependent, refractory, or intolerant of more conventional therapies has been documented. Moreover, MMF has low toxicity. Therefore, MMF may be recommended as a potential therapeutic agent for the treatment of autoimmune disease. However, the mechanism of immune suppression by MMF needs be further investigated.

In the present study, we investigated the effects of MMF in a murine model of TNBS-induced colitis via an examination of the expression of Th1- and Th2-associated cytokines and the proportion of Th1 and Th2 cells. The aim was to determine the mechanism of MMF in immune suppression and to identify the efficacy of MMF in a TNBS-induced colitic mouse model.

## 2. Results

### 2.1. MMF Ameliorates the Development of TNBS-Induced Colitis in Mice

We investigated the potential therapeutic action of MMF in a TNBS model of colitis. As expected, the mice that were administered TNBS developed a severe illness characterized by diarrhea, bloody diarrhea, rectal prolapse, and a loss of nearly 40% of their initial body weight (in 3 days); additionally 60% mortality was observed after 10 days relative to the control with only ethanol rectal administration. The mice with TNBS-induced colitis lost most of their body weight after three days, but began to recover to their original weight from day four onward. By contrast, the control mice developed no diarrhea, had reduced loss of body weight, and did not die within the same time frame.

Subsequently, we investigated whether the pretreatment of MMF can improve the clinical symptoms in TNBS-induced colitis. The survival rate of the colitic mice that were pretreated with MMF (1 or 2 mg) was significantly increased compared with that of the TNBS treatment mice (60%). Pretreatment i.p. injections of colitic mice with MMF (1 or 2 mg) resulted in an approximately 30% and 20% mortality rate, respectively. In terms of maintenance of body weight, colitic mice that were pretreated with MMF (no body weight loss was observed) were significantly different than the controls (10% of their initial body weight loss) and good performed.

**Figure 1:**
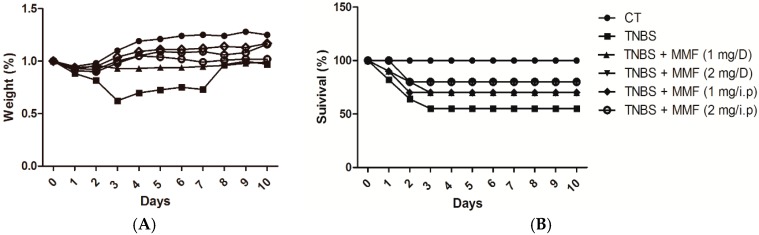
Effects of MMF treatment on the development, the survival rate and the body weight in mice with TNBS-induced colitis. Colitis was induced by the intracolonic administration of TNBS (2 mg per mouse) in 50% ethanol. Mice were treated with MMF, which was administered by i.p. or gavage (1 or 2 mg) on days −1, 0, 1, 2, and 7. Control mice received 50% ethanol alone. (**A**) The change in weight over time is expressed as the percentage of the initial body weight and was monitored daily from days 0 to 10. The administration of MMF reduced weight loss in the mice with TNBS-induced colitis; (**B**) The survival rate was recorded after the induction of colitis. Treatment with MMF significantly decreased the mortality rate in the colitic mice. MMF: Mycophenolate mofetil; TNBS: trinitrobenzene sulfonic acid-relapsing; CT: control group (only ethanol administration); D: gavage.

In another experiment, colitic mice were treated with 1 mL MMF (1 or 2 mg mL MMF) by gavage for 10 days. The mortality rates of the mice that were pretreated with 1 or 2 mg of MMF by gavage were close to 30% and 20%, respectively, which is similar to the values for the colitic mice that were pretreated with MMF by i.p. (1 or 2 mg). Mice in the first group that received pretreatment with MMF by i.p. and the colitic mice that received pretreatment with MMF (1 or 2 mg) by gavage usually maintained their initial body weight and significantly differed from the control group. The results show that pretreatment with MMF whether by i.p. injection or by gavage markedly decreased the amount of weight lost and the mortality rate in TNBS-induced colitic mice (Figure 1).

### 2.2. Histologic Changes and Inflammation Score

**Figure 2:**
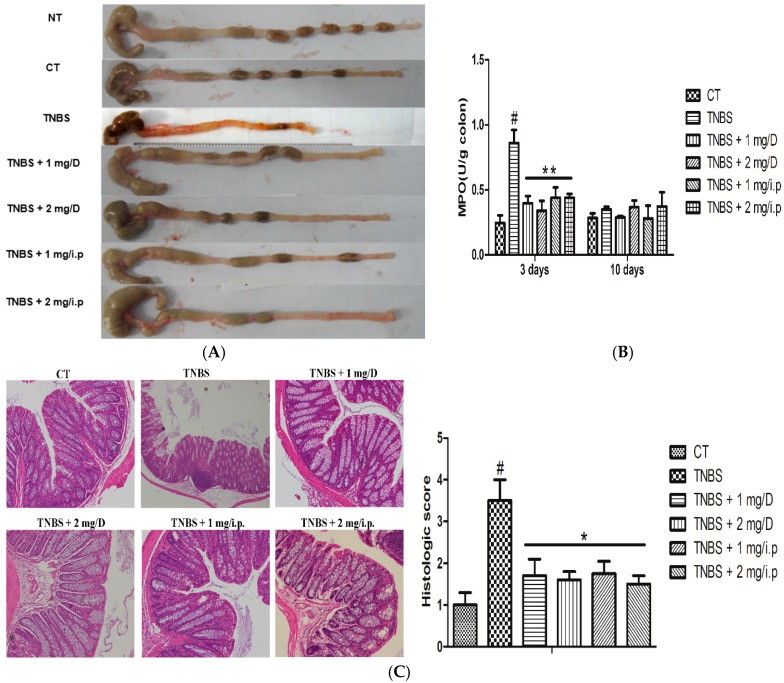
Effects of MMF treatment on the development of TNBS-induced acute colitis in mice. (**A**) Representative macroscopic images of different colons at three days after TNBS administration; (**B**) Colonic myeloperoxidase (MPO) activity was determined in the acute phase of the disease (day 3); (**C**) Three days after TNBS treatment, representative H&E-stained colon sections are shown (original magnification ×100), and the histological score was calculated. # Significant compared with the control alone, *p* < 0.05. * *p* < 0.05 and ** *p* < 0.01 *versus* the TNBS-induced colitis group.

According to previous studies, we know that weight loss and wasting disease are improved in MMF-pretreated colitic mice. In addition, macroscopically, the colon was markedly shorter in the TNBS-treated mice than in the MMF-pretreated colitic mice and the control (treated with 50% ethanol alone). This was accompanied by striking hyperemia, edema, inflammation, and necrosis compared with the control mice (Figure 2A). Histopathologically, in TNBS-treated mice group apparent reduction of goblet cells, loss of crypts, infiltration by inflammatory cells, lymphoid aggregates, focal ulcerations and/or extensive destruction of the mucosal layer were observed. However, in mice that were treated with MMF (i.p. or gavage, 1 or 2 mg), these macroscopic and histological signs were distinctly improved, which a significant reduction in the inflammatory activity and neutrophil infiltration (Figure 2C). Furthermore, histological scoring was performed as described elsewhere. The results show that the histologic scores of the TNBS-treated mice were significantly increased relative to the control group and to the MMF-pretreated colitic mice after three days (Figure 2C). In addition, myeloperoxidase (MPO) activity was correlated with neutrophil infiltration, which was obviously increased in the TNBS-treated mice compared with the control and MMF-pretreated colitic mice (i.p. or gavage, 1 or 2 mg) at three days after TNBS administration. However, the difference was not significant between the groups at 10 days (Figure 2B). In conclusion, the clinical and histological features were improved in the colitic mice that were pretreated with MMF compared with the untreated colitic mice.

### 2.3. MMF Reduced the Production of Pro-Inflammatory Cytokines in the Serum and Colon Tissues of Mice with TNBS-Induced Colitis

**Figure 3:**
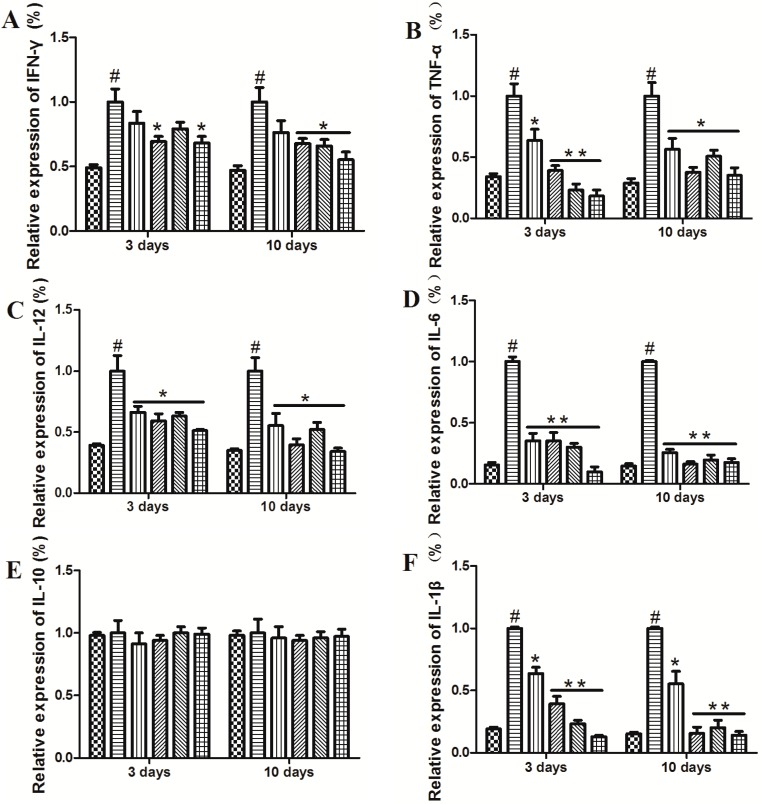

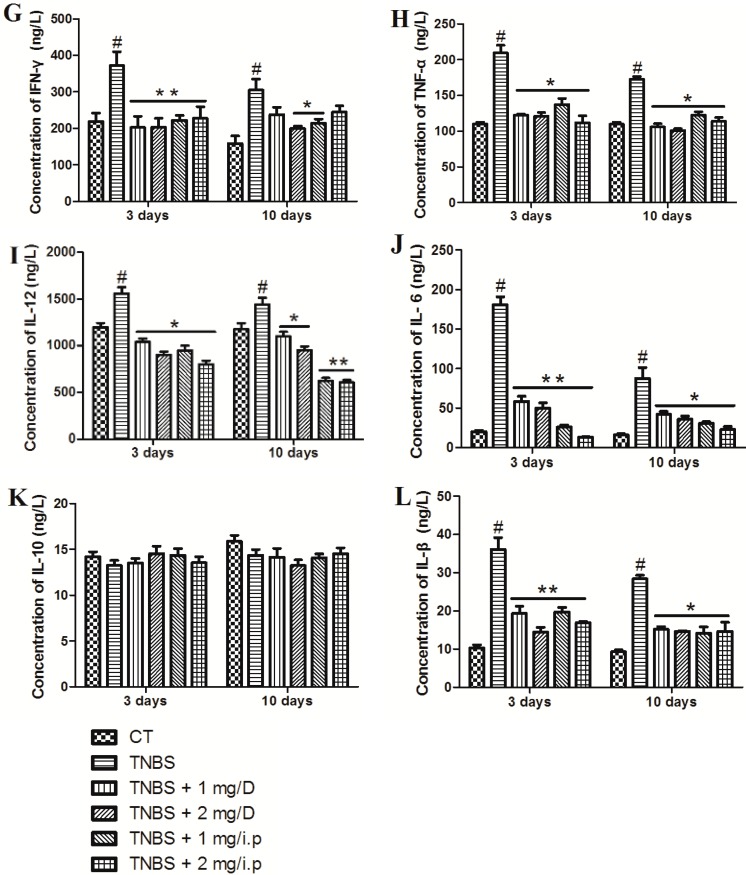
Effects of MMF on cytokine production in the serum and colon tissue samples of colitic mice. Colitis was induced by intracolonic administration of TNBS. The mice were treated with an i.p. injection of MMF (i.p. or gavage, 1 or 2 mg) 12 h after TNBS injection at day 0. Mice that were treated with ethanol alone were used as controls. At the peak of the disease (day 3) and after 10 days, the serum was collected, and total RNA was obtained from the colon. (**A**–**F**) The expression of cytokines including IFN-γ (**A**), TNF-α (**B**); IL-12 (**C**); IL-6 (**D**); IL-10 (**E**); and IL-1β (**F**) was determined by real-time PCR; (**G**–**L**) The concentrations of IFN-γ (**G**); TNF-α (**H**); IL-12 (**I**); IL-6 (**J**); IL-10 (**K**); and IL-1β (**L**) in the serum were measured by ELISA. Data represent the mean ± SEM of the fifteen mice per group. # Significant compared with the control alone, *p* < 0.05. * *p* < 0.05; ** *p* < 0.01 *versus* the TNBS-induced colitis group.

In order to investigate the mechanisms that underlie the preventive effects of MMF in mice with TNBS-induced colitis, we collected serum and colon tissue samples of mice at 3 and 10 days, respectively. When we measured the secretion of various cytokines in the serum and colon tissues, the production of cytokines including IFN-γ, TNF-α, IL-12, IL-6, and IL-1β was significantly elevated in the TNBS-treated mice at days 3 and 10. This is consistent with a previous report that showed pro-inflammatory cytokines, such as IFN-γ, IL-1β, IL-6, and TNF-a, are elevated in colonic tissues from patients with IBD [19]. Interestingly, the administration of MMF in mice with TNBS-induced colitis led to a profound decrease in the levels of IFN-γ, TNF-α, IL-12, IL-6, and IL-1β in the serum and colon tissues at days 3 and 10, while the mRNA expression levels of IFN-γ and IL-1β were markedly upregulated in colitic mice that were treated with MMF (i.p. or gavage, 2 mg) but not in those that were treated with 1 mg of MMF. Moreover, the mRNA expression level of IL-10 remained unaffected in both serum and colon tissues of the colitic mice (Figure 3). In the serum and colon tissues of patients with CD, we observed that MMF markedly decreased the levels of the pro-inflammatory cytokines IFN-γ, TNF-α, IL-12, IL-6, and IL-1β (see Figure S1). It is surprising that the levels of IL-10 mRNA were significantly increased compared with the pre-treatment levels; however, no significant difference was observed in IL-10 protein levels between the pre- and post-treatment samples (see Figure S1E,K). These results indicate that MMF has a role in the modulation of cytokine production in the setting of colonic inflammation.

### 2.4. MMF Affects the Differentiation of Splenic Th1/Th2 Cells in TNBS-Induced Colitis or in Vitro

As we know, CD is associated with a typical Th1 response, which is characterized by the production of IFN-γ and TNF-α. UC is associated with a modified Th2 response, which is dominated by IL-4, IL-6, and IL-10 [20]. The above observations indicate that the use of MMF can reduce the expression of Th1-associated molecules during experimental colitis. We thus hypothesized that MMF could directly modulate Th1 and Th2 cell differentiation. To address this issue, CD4^+^ T cells were separated from splenocytes of the control and the experimental groups of mice and then stained for IFN-γ and IL-4.

**Figure 4:**
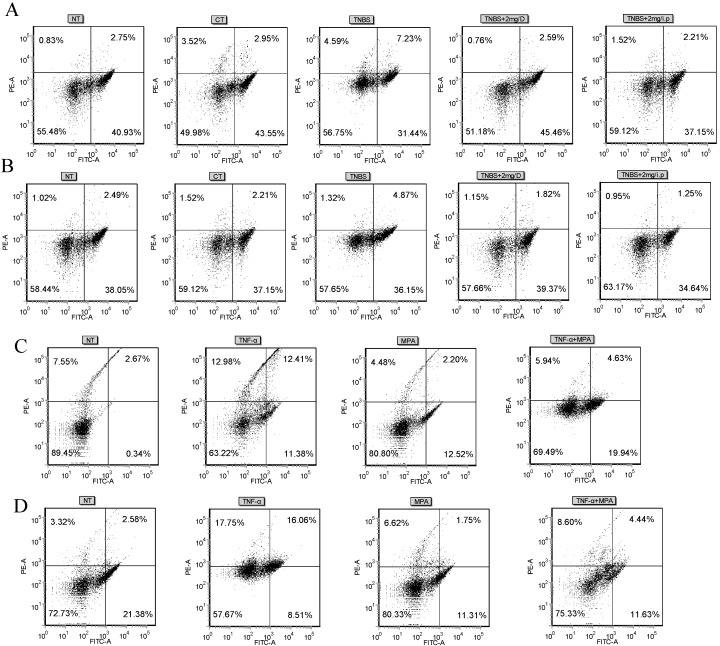
Effects of MMF on the frequency of IFN-γ or IL-4 producing CD4^+^ T cells in the colons of TNBS-treated mice. Splenocytes were isolated from normal and control TNBS-treated colitic mice and after, treatment with MMF (gavage or i.p., 2 mg) at day three. (**A**,**B**) The frequencies of CD4^+^ T cells that produce IFN-γ (**A**) or IL-4 (**B**) in the spleen were determined by flow cytometry after *in vitro* restimulation with PMA, ionomycin, and Brefeldin-A. Isolated splenocytes in normal mice were pretreated with or without MPA (50 μM) for 1 h and then stimulated with TNF-α (10 ng/mL) for 14 h. The cells were then stimulated with PMA (20 ng/mL), ionomycin (1 μg/mL), and Brefeldin-A (1 μg/mL) for 5 h; (**C**,**D**) The frequencies of CD4^+^ T cells that produce IFN-γ (**C**) or IL-4 (**D**) in splenocytes that were treated with MPA *in vitro* were determined by flow cytometry. D: gavage; PMA: phorbol 12-myristate 13-acetate; MPA: Mycophenolic acid.

Flow cytometric analysis confirmed that the percentage of CD4^+^ T cells that produce IFN-γ was markedly enhanced in the colonic splenocytes that were derived from TNBS-treated mice following stimulation with phorbol 12-myristate 13-acetate (PMA), ionomycin, and Brefeldin-A, while the administration of MMF markedly reduced the percentage of IFN-γ-producing CD4^+^ T cells. This result suggests that Th1 responses are promoted during the development of TNBS-induced colitis, while the use of MMF (gavage or i.p.) may inhibit Th1 responses (Figure 4A). Interestingly, we observed that Th2 responses were inhibited after treatment with MMF in mice with TNBS-induced colitis (Figure 4B). In parallel, in order to further elucidate whether the differentiation of Th1/Th2 cell is mediated by MMF, splenocytes were isolated from normal mice and were then stimulated *in vitro* with TNF-α in the presence or absence of MPA (50 μM). The results show that the percentage of IFN-γ-producing or IL-4-producing CD4^+^ T cells was decreased during co-treatment with TNF-α and MMF, compared with treatment with TNF-α; these data are consistent with data from colonic splenocytes that were derived from TNBS-treated mice *in vivo* (Figure 4C,D).

## 3. Discussion

Currently, the ideal therapeutic strategies for patients with IBD would induce remission and maintain long-term remission without steroid exposure and with a minimal number of surgeries [21]. Immunosuppressive drugs are quite potent agents in terms of the induction of inflammatory response remission of CD and UC. However, conventional immunosuppressive agents do not modify the disease course, but rather, they only ameliorate the symptoms and induce severe side effects, which limit their use. For example, the primary side effects of cyclosporine are renal dysfunction—which can be irreversible—neurotoxic effects, and opportunistic infections, while the side effects of methotrexate are mainly the elevation of serum aminotransferase and nausea [22]. Therefore, an effective and alternative immunosuppressive drug is urgently needed.

MMF is an immunosuppressive agent that is rapidly absorbed, completely converted to the active metabolite MPA and alternatively suppresses the proliferation of cells of the T and B cell lineage via the inhibition of DNA synthesis through its interference with purine synthesis, which is similar to the mechanisms of azathioprine [23,24]. Previous studies have reported that the immunosuppressive agent MMF has yet another immunomodulatory action *in vitro*; MMF mediates reduction of adhesion molecules glycosylation [24,25,26] and then decreases leucocyte adhesion. It was approved for the first- and second-line prevention of rejection after heterologous renal allografting [24]. Neurath *et al.*, found that MMF combined with prednisolone seems to be an effective and well-tolerated treatment for patients with active CD and who demonstrated beneficial effects after treatment with MMF that were comparable to those of azathioprine [27]. It has been reported that MMF has fewer side effects than other immunosuppressive agents such as AZA, CsA, and 5-ASA, among other. Clinically, MMF has been used for the treatment of IBD.

As we know, rectal administration of TNBS induces experimental colitis, which is characterized by severe transmural and granulomatous inflammation similar to CD in humans [28]. In this study, MMF showed highly effective remission in TNBS-induced colitis. At the onset of the disease, MMF-treated mice showed an alleviation of body weight loss, diarrhea, and intestinal inflammation and a reduction in the high mortality caused by TNBS. Additionally, we received similar results in patients with IBD who were treated with MMF. From a therapeutic point of view, it is especially important to ameliorate ongoing disease and to induce remission.

Cytokines surely play a key role in the initiation, augmentation, and perpetuation of the disease. T cell activation and excessive production of pro-inflammatory cytokines subsequently are critical factors in the pathogenesis of IBD, which highlights the ways that down-regulation of T cell activation and proliferation have potential effects in IBD [20]. Therefore, increasing numbers of patients with IBD are treated with immunomodulators such as thiopurines and anti-TNF therapy [29]. UC is characterized by a Th2 a typical immune response, with high levels of IL-6, IL-10, and IL-13, in addition to the classical pro-inflammatory cytokines [30]. CD bears all of the signs of an exaggerated CD4 T helper (Th)1 cell response, which is characterized by high TNF-α and IFN-γ. In our study, the expression of the Th1-type cytokines IFN-γ, TNF-α and IL-1β in the colon is down-regulated in response to MMF after TNBS-induced colitis in our IBD mouse model, after both three and 10 days. It appears that an inhibition of the Th1 response is generated. In contrast, the expression of the Th2-type cytokine IL-6 is down-regulated. A previous study suggested that the production of TNF-α and IFN-γ was inhibited by tacrolimus and methotrexate (MTX) in activated T cells [20]. Similarly, cortistatin dramatically reduced the protein and mRNA level of TNF-α, IFN-γ, IL-6, IL-12 and IL-1β in the mucosa of colitic mice. These findings are clearly consistent with our data. Surprisingly, in our study, the levels of the anti-inflammatory cytokine IL-10 were not significantly different. By contrast, Jienny *et al.*, found that the expression of anti-inflammatory cytokines such as IL-4 and IL-10 was not markedly changed in MMF-pretreated colitic mice relative to an untreated colitic mouse model [31]. In addition, the colons of cortistatin-treated mice showed increased expression of the anti-inflammatory cytokine IL-10 [32] while methotrexate had no significant effect on the secretion of IL-10 [33]. At the moment, we are unable to give a reason for these discrepancies, but a plausible explanation is that this difference in the cytokine production profile is also supported by the specificity of certain drugs in the treatment of either CD or UC. In patients with IBD, the production of the inflammatory cytokines TNF-α, IFN-γ, IL-6, IL-1β, IL-10 and IL-12 are in agreement with the results of the TNBS model of colitis. These results suggested that MMF can regulate the activation of inflammatory cells and cytokine production. The decrease in inflammatory mediators could lead to the amelioration of the symptoms and could induce inflammatory response remission of Crohn’s disease in the MMF-treated TNBS mice and in patients with IBD. We conclude that it is conceivable that MMF may contribute to preventing the development of inflammatory diseases by regulating the balance of Th1/Th2 cytokines.

The balance of the Th1/Th2 responses has been reported to be crucial for the development of IBD. In regards to conventional immunosuppressive agents, Th1 cytokines are more strongly inhibited by tacrolimus than Th2 cytokines while MTX generally inhibits Th1 cytokines and upregulates or does not affect Th2 cytokines, as was determined in macrophages and T cells; this alters the Th1/Th2 balance toward a Th2 profile [20]. Our data suggest that the administration of MMF markedly reduced the percentage of Th1 and Th2 cells in TNBS-induced colitis. Meanwhile, similar results were observed in splenocytes cultured *in vitro*. The ratio of Th1/Th2 responses was not obviously changed in TNBS-induced colitis, but MMF led to an immunological shift from a Th1 to a Th2 response in splenocytes cultured *in vitro*. It was shown that both Th1 and Th2 responses were inhibited by MMF. In previous reports, researchers found that the proliferation of Tregs can be inhibited by MMF in a dose-dependent manner [34], and MMF decreases the expanded B cell population through apoptosis [31]. Thus, we conclude that MMF can simultaneously inhibit humoral immunity and cellular immunity.

In conclusion, our study identifies MMF as an immunosuppressive agent with the capacity to deactivate the inflammatory response, improve histologic changes in IBD and regulate cytokine production via the modulation of Th1/Th2 cell differentiation. These data imply that MMF might be a new and promising drug for the induction and maintenance of remission in patients with IBD, and may improve therapeutic programs for patients with IBD.

## 4. Materials and Methods

### 4.1. Animals and Induction of CD

Six- to 8-week-old female BALB/c mice were purchased from the Center of Experimental Animals of Bethune Medical College of Jilin University (Jilin, China). All mice were housed in standard animal cages under specific pathogen-free conditions. The mice were maintained in an environment of constant temperature and humidity with a 12-h light-dark cycle and were given free access to food and water. All procedures and animals that were used for experimental and other scientific purposes were approved by the Protection of Vertebrate Animals. Colitis was induced in each mouse by rectal perfusion of 2 mg of TNBS (Sigma, St. Louis, MO, USA) in 50% ethanol using a vinyl catheter positioned 4 cm from the anus. Control mice received 50% ethanol alone. After the infusion, they were maintained in vertical position for 60 s. One group of colitic mice (15 mice per administration) was treated with an intraperitoneal (i.p.) injection of different concentrations (1 or 2 mg per mouse) of MMF on days −1, 0, 1, 2, and 7. A second group of colitic mice was treated with 1 mL MMF (1 or 2 mg/mL MMF) by gavage. All mice were sacrificed either 3 or 10 days after TNBS administration. Blood samples were collected by enucleation of the eyeball, and a segment of the colon was excised for evaluation of macroscopic and histological damage, cytokine determination, myeloperoxidase (MPO) activity measurement, and total RNA extraction.

### 4.2. Clinical and Histological Analysis

The animals were monitored daily for the occurrence of diarrhea, loss of body weight, and survival. Loss of body weight was determined by a calculation of the percentage of weight loss from the baseline body weight.

After death, in order to perform a histopathologic analysis, the colons were fixed, sectioned, and stained with hematoxylin/eosin; inflammation was graded from 0 to 4 as described elsewhere [32].

### 4.3. Isolation and Culture of Splenocytes

Mouse splenocytes were isolated and purified as described previously [35]. Splenocytes were obtained from control mice, those that were treated with MMF (gavage or i.p., 2 mg MMF), those with TNBS-induced colitis and from NT mice. Splenocytes were isolated and collected by centrifugation. The cells were then cultured in RPMI 1640 supplemented with 10% fetal bovine serum and 1% penicillin and streptomycin at a density of 1 × 10^7^ cells/mL in 60 mm culture dishes at 37 °C and 5% CO_2_. Two hours later, the nonadherent cells were discarded. In regards to intracellular cytokine staining, splenocytes were washed and then restimulated with phorbol 12-myristate-13-acetate (PMA) (20 ng/mL), ionomycin (1 μg/mL), and Brefeldin-A (1 μg/mL) for 5 h. In parallel, isolated splenocytes from normal mice were pretreated with or without MPA (50 μM) for 1 h, then treated with TNF-α (10 ng/mL) for 14 h. The cells were stimulated with PMA (20 ng/mL), ionomycin (1 μg/mL), and Brefeldin-A (1 μg/mL) for 5 h and harvested for flow cytometric analysis.

### 4.4. Real-Time PCR

Total RNA was extracted by Trizol reagent from mice tissue segments and was retrotranscribed into complementary DNA (cDNA). The mRNA levels of various genes were quantified using a SYBR Green QuantiTect RTPCR Kit (Roche, South San Francisco, CA, USA). β-actin was used as an endogenous reference. The primer sequences of mice were presented in Table 1:

**Table 1:** Primers of Mouse.

Gene	Sequence
β-actin sense	5′-CCAACCGTGAAAAGATGACC-3′
β-actin antisense	5′-CAGTAATCTCCTTCTGCATCC-3′
TNF-α sense	5′-CTGTGAAGGGAATGGGTGTT-3′
TNF-α antisense	5′-CAGGGAAGAATCTGGAAAGGTC-3′
IL-6 sense	5′-TATGAATAAGGCTGCTATGAA-3′
IL-6 antisense	5′-TGGTAAGGATGTGGAGAA-3′
IL-10 sense	5′-GCTCTTACTGACTGGCATGAG-3′
IL-10 antisense	5′-CGCAGCTCTAGGAGCATGTG-3′
IFN-γ sense	5′-TGAGACAATGAACGCTAC-3′
IFN-γ antisense	5′-TTCCACATCTATGCCACT-3′
IL-1β sense	5′-CTCGGCCAAGACAGGTCGCTC-3′
IL-1β antisense	5′-CCCCCACACGTTGACAGCTAGG-3′
IL-12/P40 sense	5′-TCGCAGCAAAGCAAGGTAAG-3′
IL-12/P40 antisense	5′-TGGTCTGAGGTCCAGGTGAT-3′

Gene expression was calculated relative to the housekeeping gene β-actin or to GAPDH using the ΔΔ*C*_t_ algorithm. As compared with animal, the study of CD in human can be seen in our supplementary materials.

### 4.5. ELISA Analysis

Enzyme-linked immunosorbent assay (ELISA) was used to determine the serum levels of TNF-α, IL-10, IL-1β, IFN-γ, IL-12 and IL-6 in mice according to the manufacturer’s instructions. ELISA kits were obtained from Biolegend (San Diego, CA, USA).

### 4.6. Flow Cytometry Analysis

CD4^+^ T cells were labeled with FITC-conjugated anti-mouse CD4^+^ antibodies (Biolegend) according to the manufacturer’s instructions. These cells were then stained with fluorescein-isothiocyanate (FITC)-labeled anti-mouse IFN-γ monoclonal antibody (Biolegend), and PE-labeled anti-mouse IL-4 monoclonal antibody (Biolegend) for 30 min on ice. Flow cytometric analysis was performed in a BD LSRII based on BD FACSDiva software (Becton Dickinson, CA, USA).

### 4.7. Statistical Analysis

Data were analyzed with GraphPad Prism 5 (GraphPadInStat Software, San Diego, CA, USA). Comparisons among the groups were made with ANOVA followed by Dunnett’s test. Data are presented as the mean ± SD. *p*-values of 0.05 or less were considered statistically significant (# Significant compared with the control alone, *p* < 0.05. * *p* < 0.05; ** *p* < 0.01).

## Supplementary Material

Supplementary materials can be found at http://www.mdpi.com/1422-0067/16/11/25985/s1.
