# Supplementary material for: Mycophenolate Mofetil Modulates Differentiation of Th1/Th2 and the Secretion of Cytokines in an Active Crohn’s Disease Mouse Model
Source: Int J Mol Sci. 2015 Nov 6;16(11):26654–66. doi: 10.3390/ijms161125985 (PMC4661844; doi:10.3390/ijms161125985)
Supplement: Supplementary file 1 [file ijms-16-25985-s001.pdf]

International Journal of
Molecular Sciences

MDPI

Supplementary Information

1. Materials and Methods

1.1. CD Patients and Samples

Twenty volunteer patients with active CD who were treated with MMF at the PengCheng Gastroenterology Hospital (Changchun, China) between 2011 and 2013 were enrolled in this study. Patients received administration of MMF at a dose of 1 g/day for a mean of 40 days. Blood samples were obtained from patients with IBD both before and after treatment with MMF. Data on patient demographics, erythrocyte sedimentation rate (ESR), routine blood examination (Red Blood Cell Count, RBC; hemoglobinometry; leukocyte count; neutrophilic/granulocyte count, *etc.*) were prospectively registered. Serum samples were stored at −70 °C until each participant completed the study. The levels of serum cytokines were measured by enzyme-linked immunosorbent assay (ELISA). Biopsy samples were obtained from the patients with colonic CD who underwent a colonoscopy for active disease. Colonic tissues were harvested and a portion was fixed in 4% buffered formalin, while the remaining tissue was used for total RNA extraction. The fixed tissues were embedded in paraffin, and 4–6 μm sections were stained with hematoxylin and eosin (H&E) for histological grading. The activity of disease could be slight, moderate or high. The Crohn's Disease Activity Index (CDAI) of these patients was evaluated, according to the Harvey-Bradshaw index [36,37]. This study was approved by the local ethical committees, and written informed consent was given by every patient prior to study entry.

1.2. Real-Time PCR

Biopsy samples that were obtained from patients with CD before and after treatment with MMF were frozen in liquid nitrogen and ground into a powder. Total RNA was extracted by Trizol reagent according to the manufacturer's protocol and was retrotranscribed into complementary DNA (cDNA). The mRNA levels of various genes were quantified using a SYBR Green QuantiTect RTPCR Kit (Roche, South San Francisco, CA, USA). β-actin was used as an endogenous reference. The primer sequences of human were presented in Table S1:

Table S1. Primers of Human.

Gene	Sequence
GAPDH sense	5′-ACATCATCCCTGCCTCTACTG-3′
GAPDH antisense	5′-ACCACCTGGTGCTCAGTGTA-3′
TNF-α sense	5′-AGTCCCTTAGCTGTCCCCAC-3′
TNF-α antisense	5′-TCAGGTGGCTTCCACAGAAC-3′
IL-6 sense	5′-CTTCTCCACAAGCGCCTTCGGTC-3′
IL-6 antisense	5′-TCA GGG CTG AGA TGC CGT CGA-3′
IL-10 sense	5′-AAATCGGATCTGGGGCTCTG-3′
IL-10 antisense	5′-GGAATCCCTCCGAGACACTG-3′
IFN-γ sense	5′-ACTGTCGCCAGCAGCTAAAA-3′
IFN-γ antisense	5′-GCTTAGGTTGGCTGCCTAGT-3′
IL-1β sense	5′-AGCCATGGCAGAAGTACCTG-3′

Table S1. *Cont.*

Gene	Sequence
IL-1β antisense	5′-TCCATGGCCACAACAACTGA-3′
IL-12/P40 sense	5′-GTCAGAGGGGACAACAAGGA-3′
IL-12/P40 antisense	5′-TGATGAAGAAGCTGCTGGTG-3′

Gene expression was calculated relative to the housekeeping gene β-actin or to GAPDH using the $\Delta\Delta C_t$ algorithm.

1.3. ELISA Analysis

Enzyme-linked immunosorbent assay (ELISA) was used to determine the serum levels of TNF-α, IL-10, IL-1β, IFN-γ, IL-12 and IL-6 in patients with CD before and after treatment with MMF according to the manufacturer's instructions. ELISA kits were obtained from Biolegend.

2. Results

2.1. Patient Demographics, Erythrocyte Sedimentation Rate (ESR), Routine Blood Examination and the Crohn's Disease Activity Index (CDAI)

To extend our observations to humans, we studied 20 volunteers with CD. Twenty patients (10 male, 10 female) were treated with MMF during the study period (Table S2).The age of the patients at the time the MMF treatment ranged from 24–59 years (mean age 43.8 ± 2.7 years). All of the patients suffered from CD. Of the patients with CD, 70% (14/20) experienced colonic inflammation, 20% (4/20) had ileal involvement alone, 10% (2/20) had jejunal CD, and 25% (5/20) had perianal disease. Blood samples that were obtained from the patients with CD were initially collected and analyzed for erythrocyte sedimentation rate (ESR) and routine blood examination (Table S3). The results show that the patients' routine blood examination results had changed; the number of red blood cells, neutrophils and eosinophilic leucocytes was significantly reduced compared with pre-treatment levels. However, the lymphocyte count (LYM), and the number of Mid cells (including eosinophils, basophilic granulocytes, monocytes) were not significantly different. In addition, the erythrocyte sedimentation rate (ESR) was significantly lower in patients who were treated concomitantly with MMF. Ninety percent of patients with CD were in remission (CDAI < 5), and this value was significantly lower compared with the pre-treatment value.

Table S2. Demographics of the patients with CD according to the Montreal classification.

	**CD Patients (n = 20)**
Gender: male<br>Age at diagnosis	50% (10/20)
Mean ± SE (range)	43.8 ± 2.7 year (24–59)
A1 ≤ 16	0% (0/20)
A217–40	45% (9/20)
A3 > 40<br>Disease duration	55% (11/20)

Table S2. *Cont.*

	CD Patients (n = 20)
Mean (range)<br>CD	55% (11/20)
L1-terminal ileum	20% (4/20)
L2-colon	25% (5/20)
L3-ileocolonic	45% (9/20)
L4-upper GI	10% (2/20)
P-perianal	25% (5/20)
B1-inflammatory	50% (10/20)
B2-stricturing	30%(6/20)
B3-perforating	20%(4/20)

Table S3. Routine blood examination and CDAI of patients before and after treatment with MMF.

	Before	After
RBC/L	4.1855×10^{12}	4.0765×10^{12}
Gran/L (%)	67.855	66.91
WBC/L	8.255×10^{9}	7.85×10^{9}
LYM/L (%)	20.97	25.475
HGB/L	125.9	125.35
Mid/L (%)	6.67	6.32
ESR mm/h	27.8	21.5 **
CDAI	7.7	4.9 **

RBC, red blood cells; Gran, neutrophilic granulocyte; WBC, white blood cell; LYM, lymphocyte count; HGB, hemoglobin; ESR, erythrocyte sedimentation rate; Mid, intermediate cell; CDAI, Crohn's disease activity index; Before, patients with CD before treatment with MMF; After, patients with CD after treatment with MMF. * $p < 0.05$; ** $p < 0.01$.

2.2. The Effect of MMF on the Cytokines Secreted in the Serum and Colon Tissues in Patients with CD

The aforementioned experiments showed that the administration of MMF led to a significant decrease in the levels of IFN-γ, TNF-α, IL-12, IL-6, and IL-1β in the serum and colon tissues in a TNBS model of colitis. To further confirm the role of MMF in CD, we initially harvested blood samples and biopsy samples from patients with colonic CD and then determined whether the administration of MMF could affect the course of CD in these patients. We observed that MMF markedly decreased the levels of the pro-inflammatory cytokines IFN-γ, TNF-α, IL-12, IL-6, and IL-1β in the serum and colon tissues of patients with CD (Figure S1). It is surprising that the levels of IL-10 mRNA were significantly increased compared with the pre-treatment levels; however, no significant difference was observed in IL-10 protein levels between the pre- and post-treatment samples (Figure S1E,K).

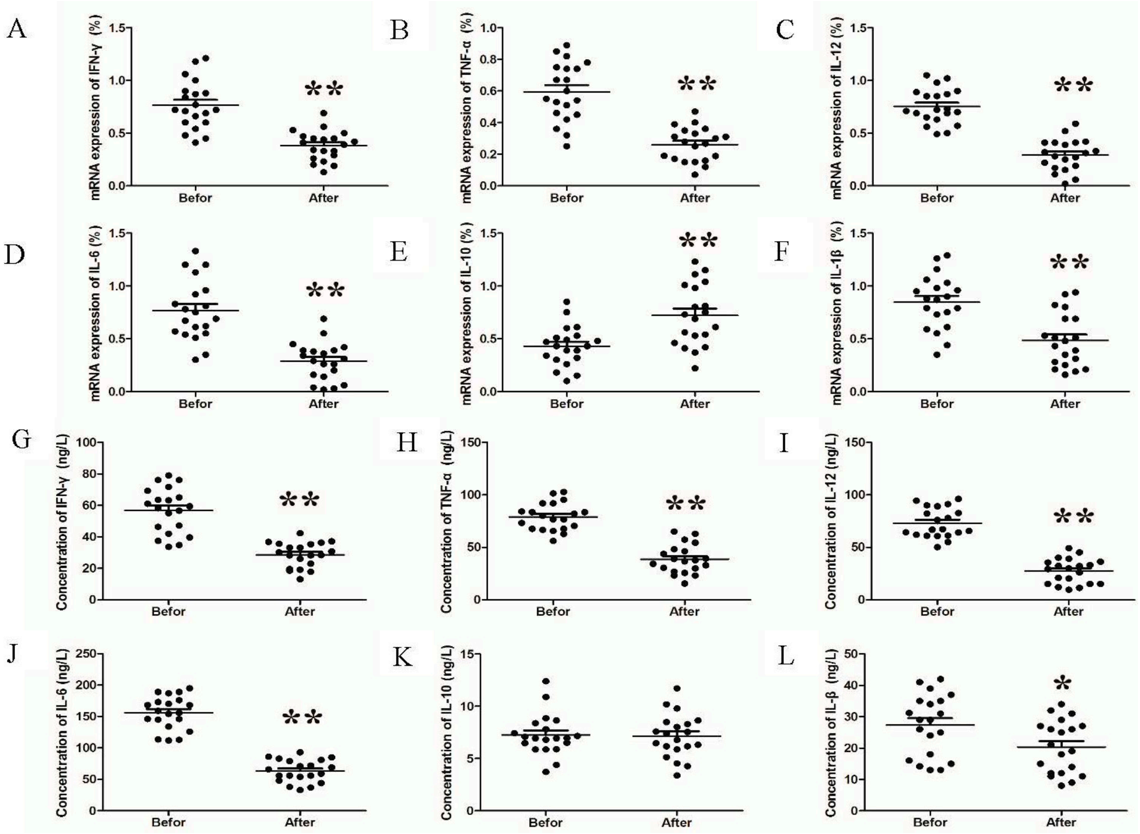

Figure S1. Effects of MMF on cytokine production in the serum and colon tissue samples from patients with CD. Patients just received administration of MMF and the duration of treatment at a dose of 1 g/day for a mean of 40 days. Then, the blood and biopsy samples were obtained from the patients with colonic CD (active disease) both before and after treatment with MMF. The serum was collected for ELISA, and total RNA was obtained from the colon tissues of patients with CD and used for the analysis of cytokine production by real-time PCR. (**A**–**F**) Gene expression of cytokines including IFN-γ (**A**); TNF-α (**B**); IL-12 (**C**); IL-6 (**D**); IL-10 (**E**), and IL-1β (**F**) was determined by real-time PCR. (**G**–**L**) The concentrations of IFN-γ (**G**); TNF-α (**H**); IL-12 (**I**); IL-6 (**J**); IL-10 (**K**); and IL-1β (**L**) in the serum were measured by ELISA. Data represent the mean ± SEM of the patients with colonic CD (active disease) both before and after treatment with MMF. * $p < 0.05$; ** $p < 0.01$ *vs.* the TNBS-induced colitis group.
